# Evaluation of Prebiotic and Health-Promoting Functions of Honeybee Brood Biopeptides and Their Maillard Reaction Conjugates

**DOI:** 10.3390/foods13172847

**Published:** 2024-09-07

**Authors:** Sakaewan Ounjaijean, Supakit Chaipoot, Rewat Phongphisutthinant, Gochakorn Kanthakat, Sirinya Taya, Pattavara Pathomrungsiyounggul, Pairote Wiriyacharee, Kongsak Boonyapranai

**Affiliations:** 1 Research Institute for Health Sciences, Chiang Mai University, Chiang Mai 50200, Thailand; sakaewan.o@cmu.ac.th; 2 Multidisciplinary Research Institute, Chiang Mai University, Chiang Mai 50200, Thailand; supakit.ch@cmu.ac.th (S.C.); rewat.p@cmu.ac.th (R.P.); sirinya.t@cmu.ac.th (S.T.); pairote.w@cmu.ac.th (P.W.); 3 Research Center of Microbial Diversity and Sustainable Utilization, Faculty of Science, Chiang Mai University, Chiang Mai 50200, Thailand; 4 Faculty of Agro-Industry, Chiang Mai University, Chiang Mai 50100, Thailand; gochakorn.g@gmail.com (G.K.); pattavara.p@cmu.ac.th (P.P.); 5 Processing and Product Development Factory, The Royal Project Foundation, Chiang Mai 50100, Thailand

**Keywords:** honeybee brood, honey, biopeptides, prebiotic, biological functions

## Abstract

This study addresses the growing interest in natural functional ingredients by evaluating the prebiotic and health-promoting functions of honeybee brood biopeptides (HBb-Bps) and their conjugates. The purpose was to investigate their antioxidant activities, enzyme inhibition properties, and effects on probiotic growth and short-chain fatty acid (SCFA) production. The HBb-Bps were conjugated with honey, glucose, and fructose via the Maillard reaction. Antioxidant activities were assessed using DPPH and ABTS assays. The inhibitory effects on amylase, pancreatic lipase, and the angiotensin-converting enzyme (ACE) were measured. Probiotic growth and SCFA production were evaluated using *L. plantarum* TISTR846, and *L. lactis* TISTR1464. The HBb-Bps and their conjugates exhibited enhanced antioxidant activities post-Maillard reaction. They showed moderate enzyme inhibition, which decreased after conjugation. However, ACE inhibition increased with conjugation. The HBb-Bps significantly promoted probiotic growth and SCFA production, with further enhancement by the Maillard reaction. Overall, the HBb-Bps and their conjugates demonstrate significant prebiotic and health-promoting functions, suggesting their potential as natural ingredients in functional foods and nutraceuticals. Further research should focus on the in vivo effects and, given their solubility and stability these biopeptides could be incorporated into functional food formulations, such as health beverages, protein bars, and other fortified foods designed to deliver specific health benefits.

## 1. Introduction

In recent years, the utilization of protein hydrolysates in health-related products has garnered significant attention due to their multifaceted biological activities and potential health benefits [1,2]. Protein hydrolysates are produced by breaking down proteins into smaller peptides and amino acids through processes such as enzymatic hydrolysis, yielding bioactive peptides with various therapeutic properties. These biopeptides (Bps) have been extensively studied for their roles in antioxidants [3,4], anti-inflammatory activities [4], antihypertensive activities [5], anti-diabetic activities [6], and anti-obesity activities [7]. The emerging trend of incorporating protein hydrolysates into functional foods, nutraceuticals, and pharmaceuticals highlights their growing importance in promoting health and well-being [8].

One intriguing source of bioactive peptides is the honeybee brood (HBb), which includes the larvae and pupae stages of bees [9]. Honeybee brood is rich in proteins, vitamins, and minerals, making it a valuable resource to produce bioactive compounds [10]. Traditionally, honeybee products such as honey, royal jelly, and propolis have been well-regarded for their health benefits [11]. However, the potential of honeybee brood, specifically its protein hydrolysates, has only recently begun to be explored [12,13,14,15]. The unique nutritional profile and bioactive properties of honeybee brood peptides make them promising candidates for developing new health-promoting products.

Biopeptides derived from honeybee broods have shown a range of biological activities that contribute to their potential as functional ingredients [13,14]. These peptides exhibit antioxidant properties, which help neutralize free radicals and reduce oxidative stress in the body [16]. Additionally, they have demonstrated anti-inflammatory effects, which can alleviate symptoms of chronic inflammation and related diseases [13]. The antihypertensive properties of these peptides are attributed to their ability to inhibit the angiotensin-converting enzyme (ACE), thereby helping to regulate blood pressure [17]. Moreover, honeybee brood peptides have been found to possess anti-diabetic and anti-obesity activities, making them valuable in managing metabolic disorders [18].

In the quest to enhance the biological properties of biopeptides, conjugation with sugars has emerged as a novel approach. The process of conjugating peptides with sugars can improve their stability, solubility, and bioavailability [19]. One effective method for achieving this conjugation is the moist-dry heating method combined with spontaneous aging [20]. This method involves heating the peptide–sugar mixture under controlled moisture conditions, followed by a period of spontaneous aging, which facilitates the Maillard reaction. The Maillard reaction is a chemical reaction between amino acids and reducing sugars that results in the formation of advanced glycation end products (AGEs) with enhanced functional and biological activities [21]. The conjugated products exhibit improved antioxidant activity [22,23], antimicrobial activity [24,25], antihypertensive activity [25], prebiotic activity [26], and antitumor effects [27]. Thus, the potential for enhancing the bioactive properties of honeybee brood biopeptides through the Maillard reaction is particularly promising.

In this study, we determined the prebiotic properties and biological activities of novel honeybee brood biopeptides (HBb-Bps), representing a promising avenue for developing innovative health-promoting products. By leveraging the unique properties of honeybee brood peptides and enhancing their bioactivity through conjugation with glucose, fructose, and honey, we can create functional ingredients with potent health benefits. This research not only expands our understanding of the therapeutic potential of protein hydrolysates but also opens new possibilities for their application in functional foods, nutraceuticals, and pharmaceuticals with an aim toward improving human health and well-being.

## 2. Materials and Methods

### 2.1. Materials and Chemicals

The honeybee brood (*Apis mellifera* L.) was harvested from a Longan honey farm located in the Maewang District in the Chiang Mai Province in Thailand. The raw bee brood was steamed using a stainless-steel steamer for 1 h and then stored in plastic bags in a freezer until further use. The longan honey used in this study was purchased from the Koonton Bee Farm in the Sansai District in the Chiang Mai Province in Thailand. The strain of *Rhizopus oligosporus* TISTR3527 (*R. oligosporus*), and 2 strains of Lactobacillus, *L. plantarum* TISTR846 (*L. plantarum*), and *L. lactis* TISTR1464 (*L. lactis*), were obtained from the Biodiversity Research Centre (BRC) at the Thailand Institute of Scientific and Technological Research (TISTR) in Pathum Thani, Thailand. All chemicals and enzymes were purchased from Sigma-Aldrich (Burlington, MA, USA).

### 2.2. Processing of Honeybee Brood Biopeptides (HBb-Bps) Preparation

The raw steamed honeybee brood was mixed and blended with deionized water at a ratio of 1:3 (*w*/*v*) for 1 min. The resulting slurry was filtered through a 10 μm nylon bag to obtain a honeybee brood solution. Sodium hydroxide solution was then added to the solution until the pH reached 8, and the mixture was centrifuged at 7500 rpm at 4 °C for 10 min, resulting in a protein precipitate. This precipitate product was defatted by agitating it with hexane at a 1:10 (*w*/*v*) ratio for 3 h with gentle shaking. The hexane was removed from the sediment through filtration, and the sediment was dried overnight at room temperature, yielding a honeybee brood protein powder.

The HBb-Bps were prepared through the hydrolysis of honeybee brood protein powder using a fungal fermentation technique [28]. Firstly, the moisture content of the protein powder was adjusted to 45–50% before being autoclaved at 115 °C for 15 min. The protein was then incubated with 10% of *R. oligosporus* (10^6^ spores/mL) for solid-state fermentation at 25 °C for 48 h. The product was subjected to freeze-drying (Gamma 2–16 LSCplus, CHRIST, Osterode am Harz, Germany) at a temperature of −80 °C and a pressure of 0.1 mbar. After freeze-drying, the powder was ground using a mechanical grinder and passed through a 100-mesh sieve to achieve a uniform particle size. The peptide content of the HBb-Bps was determined using a Kjeldahl analysis, with a peptide concentration of approximately 82 g per 100 g on a dry basis. The resulting HBb-Bps powder was stored at −18 °C in a polyethylene bag for further use.

### 2.3. Preparation of HBb-Bps Conjugated Products Using the Moist-Dry Heating Method with Spontaneous Aging Technique

An interaction between HBb-Bps and glucose, fructose, and honey was conducted via the Maillard reaction using a moist-dry heating method as previously described [20]. The HBb-Bps were prepared at a concentration of 1% (*w*/*v*) in 100 mL of deionized water. This HBb-Bps solution was subsequently combined in a 1:1 (*v*/*v*) ratio with 1% (*w*/*v*) solutions of glucose, fructose, or honey. Each mixture was aged in a desiccator under a controlled relative humidity of 75% at 60 °C for 20 days. The aging process was terminated by freezing the samples at −18 °C. After the Maillard process, the high molecular weight (1.0–10.0 kDa) of the HBb-Bp glucose, HBb-Bp fructose, and HBb-Bp honey increased to 36%, 66%, and 67%, respectively. The solubility tests showed that at pH 7, both the HBb-Bp powder and the HBb-Bp conjugated products exhibited excellent solubility, with more than 95% of the substances dissolving in water. After which, they were subjected to biological characterization analyses.

### 2.4. Characterization of HBb-Bps and HBb-Bp Conjugated Products

#### 2.4.1. Degree of Protein Hydrolysis

The degree of hydrolysis was determined using a modified formal titration method based on the procedure outlined in [29]. For this analysis, approximately 1.5 to 1.7 g of protein hydrolysate samples were mixed with 45 to 50 mL of purified water. The pH of the mixture was adjusted to 7.0 using a 0.1 N NaOH solution. Next, 10 mL of 37% (*v*/*v*) formaldehyde was added, and the mixture was allowed to stand for 8 min at room temperature. Titration was then performed to reach an endpoint of pH 8.5, using a 0.1 N NaOH standard solution. The amount of free amino groups was calculated based on the volume of NaOH consumed during titration. Total nitrogen content was measured using the Kjeldahl method [15]. The degree of hydrolysis and the concentration of free amino groups were subsequently calculated as follows:

Free amino groups (%) = (V × C × 14.007/M × 1000) × 100

Degree of hydrolysis (DH) = (% Free amino groups/% total nitrogen) × 100

where V = volume consumed of NaOH (mL); C = the concentration of NaOH (0.1 M); and M = the quantity of hydrolysate in the sample (g).

#### 2.4.2. Molecular Weight Distribution Using Size-Exclusion HPLC

The molecular weight distribution and peptide length were assessed using size-exclusion high-performance liquid chromatography (SEC-HPLC) [30]. This setup employed a UV–VIS detector set to 280 nm and an SRT-C SEC-300 column (5 μm, 7.8 × 300 mm, Sepax Technologies, Inc., Newark, DE, USA). The column was pre-equilibrated and eluted with a 0.1 M sodium phosphate buffer at pH 7.0, running in isocratic mode at a flow rate of 1.0 mL/min, while the column temperature was maintained at 30 °C. Samples were dissolved in deionized water, filtered through a 0.45 μm membrane, and injected at a volume of 15 μL. The calibration curve was generated using a protein standard mix ranging from 15 to 600 kDa, including bovine thyroglobulin (~670 kDa), bovine gamma-globulins (150 kDa), chicken egg albumin (44.3 kDa), bovine pancreas ribonuclease A (13.7 kDa), and p-aminobenzoic acid (pABA) as a low molecular weight marker. This calibration curve was constructed by plotting the logarithmic molecular weight of the standards against their elution times. The relative molecular size distribution was then estimated by integrating the area under the resulting chromatograms.

#### 2.4.3. Determination of Amino Acid Composition by HPLC

An amino acid profiling was carried out using a post-column reaction method as outlined by Somjai et al. [31]. The analytical system utilized a Shim-pack AMINO-NA column (100 mm in length × 6.0 mm internal diameter, with a 5 μm particle size, part number 228-18837-91, Shimadzu, Kyoto, Japan), coupled with a Prominence RF-20A fluorescence detector from Shimadzu, Japan. The mobile phases included sodium citrate buffers, labeled as A, B, and C, with pH values of 3.23 (A) and 10.0 (B), while phase C consisted of a 0.2 M NaOH aqueous solution. Amino acids underwent pre-column derivatization using N-Acetyl-L-cysteine and OPA as reaction reagents. The method operated under the conditions of a 0.4 mL/min flow rate, a column oven temperature of 60 °C, and a sample injection volume of 10 µL. All samples analyzed in this study were assessed for their amino acid content.

### 2.5. Biological Properties of HBb-Bps Interaction with Sugar and Honey

#### 2.5.1. Antioxidant Activities of HBb-Bps Interaction with Sugar and Honey

The HBb-Bps, sugar, honey, and their conjugates were evaluated for their antioxidative properties using two distinct methods: the DPPH radical scavenging assay, and the ABTS radical cation assay, following the established protocols

The free radical scavenging ability of the samples was tested by the DPPH radical scavenging assay as described in [32]. In brief, a solution of 0.1 mM DPPH in Methanol was prepared, and 2.4 mL of this solution was mixed with 1.6 mL of each sample. The reaction mixture was vortexed thoroughly and left in the dark at room temperature for 30 min. The absorbance of the mixtures was measured spectrophotometrically at 517 nm. A calibration curve was constructed using Trolox, and the antioxidative activity was represented by the percentage inhibition of radical scavenging.

The free radical scavenging capacity of the samples was studied using the ABTS radical cation decolorization assay as previously described [33]. Initially, an oxidizing solution was prepared by mixing 2.45 mM potassium persulfate with 7 mM ABTS solution in a 20 mM sodium acetate buffer (pH 4.5). This mixture was incubated at room temperature for 12–16 h to achieve a stable, dark blue-green radical solution. The ABTS radical cation (ABTS+·) was diluted with 95% ethanol until it reached an absorbance of 0.70 ± 0.02 at 734 nm, forming the working solution. Then, 100 μL of sample solution was added to 3 mL of the working solution, and the absorbance was measured at 734 nm after incubating the solution at 30 °C, in darkness for 10 min. The ABTS radical inhibition capacity was determined using a Trolox calibration curve.

#### 2.5.2. Inhibition of α-Amylase Activity

The α-amylase inhibitory activity of the sample was measured using a slightly modified approach as previously described [32]. The sample solution was prepared by dilution with a 100 mM phosphate buffer with pH 7.0. The reaction mixture containing 50 μL of the sample solution, 10 μL of the phosphate buffer, and 20 μL of the porcine pancreas amylase (A3176) was pre-incubated at 37 °C for 5 min. Then, 20 μL of 1% soluble starch was added as the substrate, and the mixture was incubated at 37 °C for an additional 5 min. The reaction was stopped by adding 50 μL of 1 M HCl and 50 μL of iodine solution. As a positive control, acarbose was utilized. The absorbance was measured at 650 nm., and the percentage of enzyme inhibitory rate was calculated using the formula: α-amylase inhibition (%) = 100 − [(A_0_ − A_S_) × 100/(A_0_ − A_NC_)] × 100 where A_0_ is the absorbance of the blank (without an enzyme), A_NC_ is the absorbance of the control (without an inhibitor) and A_S_ represents the absorbance of the sample or positive control.

#### 2.5.3. Inhibition of Pancreatic Lipase Activity

The anti-hyperlipidemia assay, which is based on the in vitro inhibition of pancreatic lipase activity, was conducted following the method outlined in a previous study [32]. In brief, the sample solution was prepared by dilution with a 100 mM phosphate buffer with pH 7.0. Then, 10 μL of the solution was mixed with 5 μL of 50 mM 4-nitrophenyl butyrate (p-NPB) and 175 μL of the phosphate buffer. Subsequently, 10 μL of Type II Lipase from the porcine pancreas was added, and the reaction mixture was allowed to proceed at 37 °C for 30 min. Orlistat was used as a positive control. The absorbance of the reaction mixture was measured at 410 nm. The percentage of enzyme inhibitory rate was calculated using the formula: Pancreatic lipase inhibition (%) = [A_NC_ − A_S_/A_NC_] × 100 where A_NC_ is the absorbance of the control (without an inhibitor) and A_S_ is the absorbance of the sample or positive control.

#### 2.5.4. Inhibition of Angiotensin-Converting Enzyme Activity (ACE)

The ACE inhibition was carried out using the colorimetric method via the ACE kit-WST (Dojindo Inc., Kumamoto, Japan). In brief, 20 μL of a sample solution (1–100 mg/mL), Enalapril (ACE inhibitor, used as the positive control), and deionized water (as a blank) were added to the well of a 96-well plate. Subsequently, 20 μL of substrate buffer was added to each well, followed by the addition of 20 μL of an enzyme working solution. The reaction mixtures were incubated at 37 °C for 60 min. After incubation, 200 μL of the indicator working solution was added to each well and further incubated at 37 °C for 10 min. The absorbance was measured at 450 nm. The percentage of enzyme inhibitory rate was calculated using the formula: ACE inhibition (%) = [(A_C_ − A_S_)/(A_C_ − A_BL_)] × 100 where A_C_ is the absorbance of the control (without an inhibitor) and A_S_ is the absorbance of the sample or positive control. A_BL_ is the absorbance of the blank.

### 2.6. Prebiotic Properties of HBb-Bps Interaction with Sugar and Honey

#### 2.6.1. Prebiotic Effect on Selected *Lactobacillus* spp. Growth

The prebiotic effect of HBb-Bps conjugated with honey was evaluated through the *L. plantarum* and *L. lactis* growths. Initially, the bacterial strains were activated by incubation in a Man–Rogosa–Sharp (MRS) broth at 37 °C for 1 day before use. Then, the bacteria solution (1% (*v*/*v*)) was inoculated to the MRS broth supplied with 1% (*w*/*v*) dextrose as a reference carbon source. The HBb-Bps, HBb-Bps conjugated with honey, inulin, and Fructo-oligosaccharide (FOS) that replaced the dextrose were used as a supplemented carbon source at a concentration of 1% (*w*/*v*). The MRS without dextrose or sample served as the blank. All cultures were incubated at 37 °C and aliquots of the fermentation broth (5 mL) were obtained at 0, 3, 6, 9, 12, 24, 36, and 48 h for optical density (OD) and pH determination. The prebiotic index (PI) was calculated by the following equation [30]:

PI = [A_S48_ − A_S0_ − (A_BL48_ − A_BL48_)]/[A_D48_ − A_D0_ − (A_BL48_ − A_BL48_)] (1)

where A_S0_ and A_S48_ denote the OD value of the culture containing samples or FOS as a carbon source at 0 and 48 h; A_D0_ and A_D48_ are the OD of the culture with dextrose as a carbon source at 0 and 48 h; A_BL0_ and A_BL48_ are the OD of the culture without a carbon source at 0 and 48 h.

#### 2.6.2. Quantification of Short-Chain Fatty Acids (SCFA)

The production of lactate and SFCAs (acetate, propionate, and butyrate) was evaluated according to Alvarez et al. (2023) [34], using HPLC (LC-40D, Shimadzu) fitted with a refractive index detector (RID-20A, Shimadzu). For this purpose, an Aminex HPX87H column (300 mm × 7.8; Biorad, Hercules, CA, USA) was employed at 50C using a mobile phase of 3 mM sulfuric acid in an isocratic model at a flowrate of 0.6 mL/min.

### 2.7. Statistical Analysis

The results are presented as the mean + standard derivation (SD) obtained from three independent experiments. Statistical analyses were performed using a one-way ANOVA with SPSS version 20 (IBM, Armonk, NY, USA). Duncan’s multiple range test was applied to find significant discrepancies between the mean values. Finally, *p* < 0.05 was used as the criterion of significance.

## 3. Results

### 3.1. Antioxidant Activities of HBb-Bps, Honey, and Sugar and Their Conjugating Products before and after the Moist-Dry Heating Process

During the preparation of honeybee brood biopeptides (HBb-Bps) using solid-state fermentation with 10% Rhizopus oligosporus (10^6^ spores/mL), it was observed that the degree of hydrolysis of the honeybee brood proteins peaked at 70% after 48 h of fermentation. Further analysis of the hydrolysates using high-performance liquid chromatography (HPLC) indicated that the peptides produced were distributed across various molecular weight ranges: approximately 18% were >0.1 kDa, 61% were between 0.10 and 1.0 kDa, and 21% were between 1.0 and 10.0 kDa. Amino acid profiling revealed that the hydrolysates predominantly contained valine (17%), alanine and cysteine combined (17%), phenylalanine (13%), and glutamic acid (13%), with other amino acids present at less than 10%.

**Figure 1:**
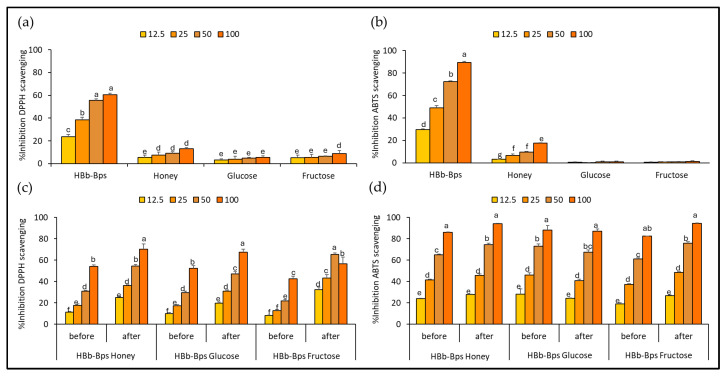
Antioxidative activities by (**a**) DPPH and (**b**) ABTS scavenging assay of individual HBb-Bps, honey, glucose, and fructose solutions at 12.5, 25, 50, and 100 mg/mL. (**c**) DPPH, and (**d**) ABTS scavenging assay of the combination between HBb-Bps and honey, glucose, or fructose solutions at 12.5, 25, 50, and 100 mg/mL before and after interaction through spontaneous aging for 20 days under a moist-dry heating process. Different lowercase letters indicate significant differences among the different concentrations (*p* < 0.05).

The antioxidant activities of individual substrates for conjugation, HBb-Bps, honey, glucose, and fructose, were evaluated using the DPPH assay (Figure 1a) and ABTS assay (Figure 1b). The results are reported as the percentage inhibition of DPPH scavenging or ABTS scavenging at four different concentrations: 12.5, 25, 50, and 100 mg/mL. These findings indicate that HBb-Bps exhibited the highest antioxidant activities across all concentrations, with a percentage inhibition increasing from 23.53% ± 2.28 at 12.5 mg/mL to nearly 60.72% ± 2.41 at 100 mg/mL for DPPH assay, and 29.55% ± 0.93 at 12.5 mg/mL to nearly 89.30% ± 1.09 at 100 mg/mL for ABTS assay. Statistical analysis shows significant differences among the concentrations, with higher concentrations of HBb-Bps displaying markedly greater antioxidant activity. Honey demonstrated moderate antioxidant activities, with inhibition ranging from 5.70% ± 2.48 to 13.15% ± 2.53 for DPPH assay, and 3.25% ± 0.24 to 17.66% ± 0.36 for ABTS assay. Unsurprisingly, glucose and fructose showed relatively low antioxidant activities, with percentage inhibition remaining below 10% at all concentrations. These results demonstrate that HBb-Bps have superior antioxidant properties compared to honey, glucose, and fructose, with significant dose-dependent effects observed in both DPPH and ABTS assays.

To investigate the outcome of conjugation by the Maillard reaction through the moist-dry heating process with the spontaneous aging technique, the antioxidant activities of the three conjugated products, HBb-Bp Honey, HBb-Bp Glucose, and HBb-Bp Fructose, were assessed using the DPPH (Figure 1c) and ABTS (Figure 1d) radical scavenging assay. The results of both before and after the Maillard reaction, at four different concentrations (12.5, 25, 50, and 100 mg/mL), were presented. The DPPH scavenging activity significantly increased for all conjugated products after the Maillard reaction. For the HBb-Bp Honey before the reaction, the percentage inhibition ranged from 11.56% ± 0.66 at 12.5 mg/mL to about 54.19% ± 1.38 at 100 mg/mL. After the reaction, inhibition improved, ranging from 25.13% ± 1.01 at 12.5 mg/mL to nearly 70.35% ± 4.79 at 100 mg/mL. Nearly 100% of inhibition was found in the ABTS result, where inhibition of HBb-Bp Honey (100 mg/mL) from 85.96% ± 0.55 before the reaction increased to 94.24% ± 0.24, after the reaction. For the HBb-Bp Glucose and HBb-Bp Fructose, both showed a similar trend as HBb-Bp Honey, with inhibition percentages increasing significantly after the Maillard reaction. These results exhibit that the Maillard reaction through the moist-dry heating process with spontaneous aging technique significantly enhances the antioxidant properties of the HBb-Bp conjugated products when measured by the DPPH and ABTS assay. However, while the Maillard reaction did enhance the DPPH scavenging activity, it was relatively modest compared to the ABTS result. This suggests that the Maillard reaction may have a variable impact on the antioxidant properties of different conjugated products.

### 3.2. Anti-Obesity Activities of HBb-Bps, Honey, and Sugar and Their Conjugating Products before and after the Moist-Dry Heating Process

**Figure 2:**
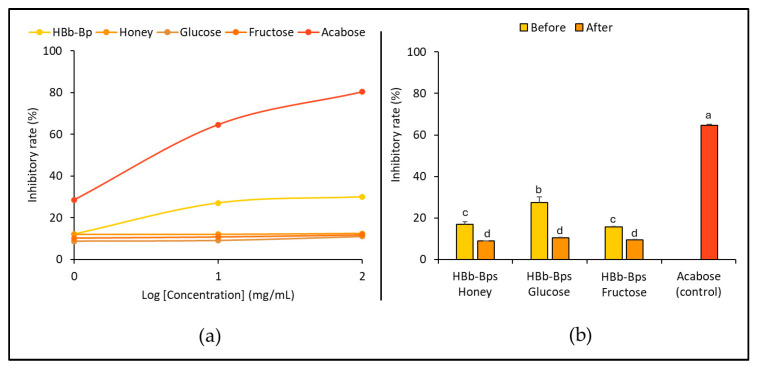
In vitro anti-obesity properties by (**a**) inhibition of α-amylase activity of individual HBb-Bps, honey, glucose, fructose, and acarbose (positive control) solutions at 1, 10, and 100 mg/mL. (**b**) inhibition of α-amylase activity of the combination of HBb-Bps and honey, glucose, or fructose solutions at 10 mg/mL before and after interaction through spontaneous aging for 20 days under a moist-dry heating process, compared to 10 mg/mL of acarbose as positive control. Different lowercase letters indicate significant differences among the different concentrations (*p* < 0.05).

The biological properties of the individual substrates and their conjugated products before and after the Maillard reaction were evaluated. Figure 2a shows the inhibition of α-amylase activity for HBb-Bps, honey, glucose, and fructose incorporated with acarbose, used as the positive control, at three different concentrations (1, 10, and 100 mg). The results indicate that the HBb-Bps exhibited moderate inhibition, with a gradual increase in the inhibition rate from 9.83% ± 0.96 at the lowest concentration to around 29.99% ± 0.09 at the highest concentration. Honey demonstrated a relatively low inhibition rate, reaching up to about 12.40% ± 0.18 at higher concentrations. Similarly, glucose and fructose exhibited a minimal inhibitory function on α-amylase, with inhibition rates remaining low across all tested concentrations, barely exceeding 12%. The data suggest that among the tested subjects, HBb-Bps show some potential for α-amylase inhibition. Honey, glucose, and fructose, however, have minimal inhibitory effects.

An effect of the Maillard reaction on the inhibition of α-amylase for the three conjugated products was investigated. HBb-Bp Honey, HBb-Bp Glucose, and HBb-Bp Fructose, both before and after the reaction, at a concentration of 10 mg/mL, were employed (Figure 2b). The results demonstrate that all three HBb-Bp conjugates showed an analog trend in inhibitory effect before and after the moist-dry heating process. Before the reaction, the inhibitory rate of the HBb-Bps Honey was approximately 16.97% ± 1.33 and slightly decreased to 8.99% ± 0.05 after the process. HBb-Bp Glucose also slightly decreased from 27.49% ± 2.71 to 10.35% ± 0.41 after the process and HBb-Bp Fructose reduced from 15.68% ± 1.65 to 9.38% ± 0.09. These results imply that the Maillard reaction resulted in a slight decrease in the amylase inhibitory effect for all three conjugates. This identical result suggests a consistent impact of the Maillard reaction on their inhibitory properties.

**Figure 3:**
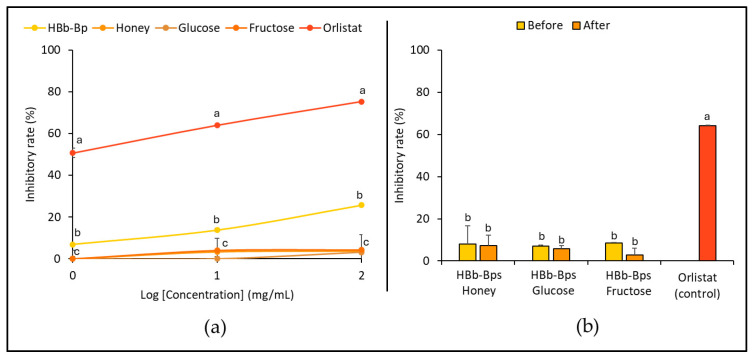
In vitro anti-hyperlipidemia properties by (**a**) inhibition of pancreatic lipase activity of individual HBb-Bps, honey, glucose, fructose, and orlistat (positive control) solutions at 1, 10, and 100 mg/mL. (**b**) inhibition of pancreatic lipase activity of the combination of HBb-Bps and honey, glucose, or fructose solutions at 10 mg/mL before and after interaction through spontaneous aging for 20 days under a moist-dry heating process, compared to 10 mg/mL of orlistat as positive control. Different lowercase letters indicate significant differences among the different concentrations (*p* < 0.05).

The inhibitory activity of pancreatic lipase, an enzyme responsible for fat digestion, was evaluated for the HBb-Bps, honey, glucose, and fructose. The inhibition tests were conducted at three different concentrations (1, 10, and 100 mg/mL) with orlistat serving as the positive control (Figure 3a). The results represent that the HBb-Bps have moderate inhibitory activity, with the inhibition rate increasing from 6.90% ± 0.14 at 1 mg/mL to 25.70% ± 0.90 at 100 mg/mL. This indicates a significant, dose-dependent increase in inhibitory activity. Honey demonstrated relatively low inhibitory activity, while glucose and fructose exhibited negligible effects, with inhibition rates remaining close to 0% across the tested concentrations. The data suggest that biopeptides derived from honeybee brood have a moderate inhibitory effect on pancreatic lipase.

Subsequently, the HBb-Bp Honey, HBb-Bp Glucose, and HBb-Bp Fructose, both before and after the Maillard reaction, at a concentration of 10 mg/mL, were employed to determine their inhibitory activity against a pancreatic lipase. Orlistat was used as the positive control. Figure 3b indicates that the inhibition rate of the three HBb-Bp conjugates showed a similar pattern before and after the Maillard reaction. All three subjects displayed an inhibition rate of approximately 10% before the reaction. After the reaction, there was a slight decrease, maintaining around the same inhibition rate with no significant change. On the other hand, orlistat showed a significantly higher inhibitory rate of about 64.00% ± 0.25, indicating its strong efficacy as a pancreatic lipase inhibitor. These results suggest that the Maillard reaction, through the moist-dry heating process, did not substantially alter the inhibitory activity of HBb-Bp Honey, HBb-Bp Glucose, and HBb-Bp Fructose.

### 3.3. Anti-Hypertensive Activity of HBb-Bps, Honey, and Sugar and Their Conjugating Products before and after the Moist-Dry Heating Process

**Figure 4:**
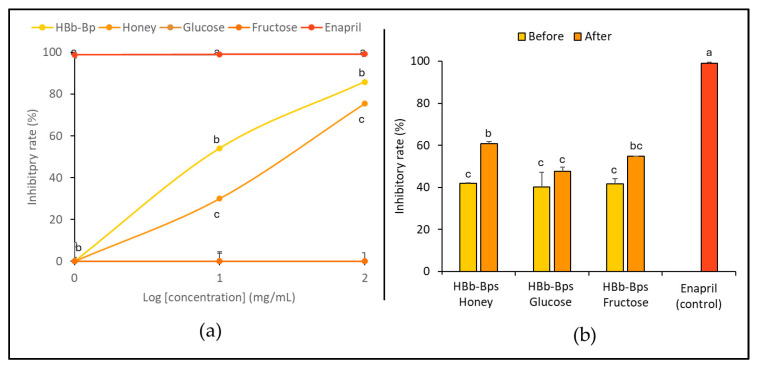
In vitro anti-hypertensive property by (**a**) inhibition of ACE activity of individual HBb-Bps, honey, glucose, fructose, and enalapril (positive control) solutions at 1, 10, and 100 mg/mL. (**b**) Inhibition of ACE activity of the combination of HBb-Bps and honey, glucose, or fructose solutions at 10 mg/mL before and after interaction through spontaneous aging for 20 days under a moist-dry heating process, compared to 10 mg/mL of enalapril as positive control. Different lowercase letters indicate significant differences among the different concentrations (*p* < 0.05).

The inhibition of the angiotensin-converting enzyme (ACE), an enzyme associated with blood pressure regulation, was evaluated. HBb-Bps, honey, glucose, and fructose were employed at three different concentrations (1, 10, and 100 mg/mL), with enalapril serving as the positive control. Figure 4a exhibits the significant inhibitory activity of HBb-Bps, with the inhibition rate increasing from 3.64% ± 2.31 at 1 mg/mL to 85.82% ± 0.14 at 100 mg/mL. Likewise, honey demonstrated moderate inhibitory activity in a dose-dependent manner, ranging from 1.64% ± 1.80 at 1 mg/mL to 75.47% ± 0.35 at 100 mg/mL. On the other hand, glucose showed minimal inhibitory activity, with rates remaining below 1% across all concentrations. Unsurprisingly, enalapril, the commercial ACE inhibitor, exhibited nearly 100% inhibition across all tested concentrations. Taken together, HBb-Bps emerged as powerful ACE inhibitors, while fructose and honey also showed significant activity, making them promising natural ACE inhibitors. Furthermore, the ACE inhibitory activity of the three conjugated products was determined. Both before and after the Maillard reaction, at 10 mg/mL, inhibitory activity tests were performed with enalapril serving as the positive control (Figure 4b). The results show that the Maillard reaction had a noticeable impact on the inhibitory activity of the conjugated products. All three conjugates displayed a similar pattern, with an increasing inhibition rate after the reaction, in which the HBb-Bp Honey increased from 42.00% ± 0.21 to 60.79% ± 0.97, the HBb-Bp Glucose increased from 40.21% ± 7.01 to 47.69% ± 1.87, and the HBb-Bp Fructose increased from 41.56% ± 2.64 to 54.71% ± 0.00. Significant changes were found in the conjugating products of the HBb-Bp Honey and HBb-Bp Fructose. These findings highlight the potential of Maillard reaction products, particularly HBb-Bp Honey and HBb-Bp Fructose, as effective ACE inhibitors.

### 3.4. Prebiotic Properties of HBb-BPs, HBb-Bp Honey, and Commercial Prebiotic Substances

**Figure 5:**
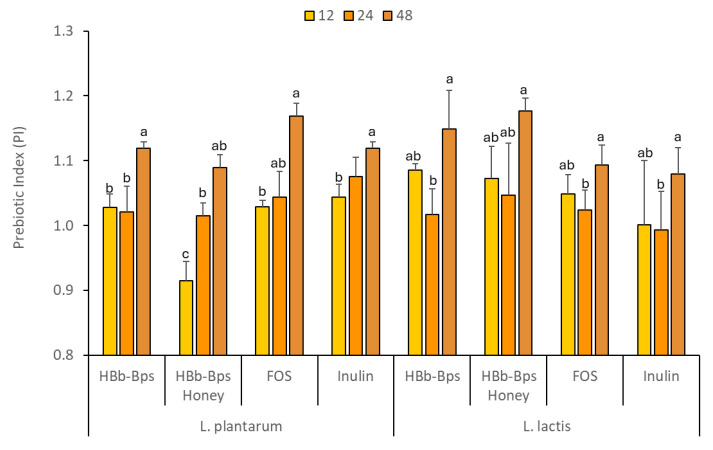
Prebiotic index (PI) values at 12, 24, and 48 h of *L. plantarum*, and *L. lactis* fermentation in the prebiotic-enriched media (HBb-Bps, HBb-Bp honey, FOS, and inulin). The result represents the average of two independent fermentations and triplicate analysis of each sample ± standard deviation. Data represented as mean ± SD (n = 3); lowercase letter (a–c) indicate values within each row with different superscript letters were significantly different (*p* < 0.05).

The prebiotic properties of HBb-Bps, HBb-Bp Honey, FOS, and inulin were evaluated by comparing the growth of probiotic bacteria and changes in pH when these substances replaced dextrose as the carbon source. This study was conducted at a concentration of 1% (*w*/*v*) for each test substance, with 1% (*w*/*v*) dextrose as the control. The effects on two Thai probiotic strains, L. plantarum, and L. lactis, were observed over an incubation period of 48 h (Table 1). The growth of L. plantarum, measured as OD620, showed that HBb-Bps, Hbb-Bp Honey, FOS, and inulin significantly promoted bacterial growth compared to the control. At 48 h, the FOS showed the highest growth (3.23 ± 0.06), followed by HBb-Bp Honey (3.22 ± 0.02). HBb-Bp Honey and inulin also supported bacterial growth but to a lesser extent than the FOS and HBb-Bps. For L. lactis, the growth patterns were comparable to those observed in L. plantarum. All prebiotic substances significantly supported bacterial growth compared to the control. HBb-Bp Honey promoted the highest bacterial growth (3.01 ± 0.10) at 48 h, followed closely by HBb-Bps (3.00 ± 0.01). FOS and inulin supported moderate growth, higher than dextrose but lower than the HBb-Bp and its conjugate. As a result, the prebiotic index (PI) of HBb-Bps, HBb-Bp Honey, FOS, and inulin for both bacterial strains were similarly observed (Figure 5). The significant change in the prebiotic index (PI) at 48 h compared to 12 and 24 h, as observed in Figure 5, and can be attributed to the time-dependent nature of microbial fermentation and growth. During the initial 12 to 24 h period, the growth of probiotics such as *L. plantarum* and *L. lactis,* in the media rich in HBb-Bps, FOS, and inulin, reaches a steady state, with similar PI values. However, by 48 h, the extended incubation period allows for further fermentation and metabolism, leading to a significant increase in the production of short-chain fatty acids (SCFAs) and other metabolites. This prolonged fermentation can enhance the utilization of prebiotic substrates, resulting in a higher prebiotic index. Thus, these findings exhibit the potential of the biopeptides from honeybee brood and their conjugates to be used as effective prebiotics, which could be beneficial for gut health.

**Figure 6:**
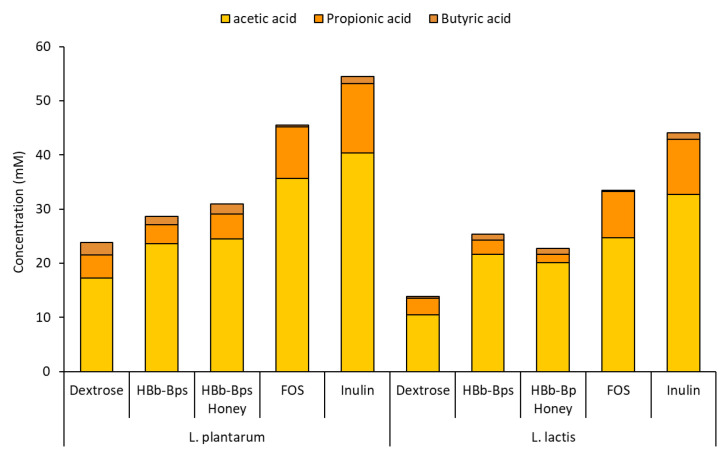
Production of SCFA (acetic acid, Propionic acid, and butyric acid) after 48 h of *L. plantarum* and *L. lactic* fermentation in the control carbon source (dextrose) or prebiotic-enriched media (HBb-Bps, HBb-Bp honey, FOS, and inulin). The result represents the average of two independent fermentations and triplicate analysis of each sample ± standard deviation.

These experiments assessed the impact of different carbon sources on the pH changes and production of short-chain fatty acids (SCFAs). The pH of the cultures was monitored over 48 h (Table 1). For both *L. plantarum* and *L. lactis*, the initial pH ranged from 5.28 to 5.42. As fermentation progressed, the pH decreased significantly for all carbon sources, indicating acid production. After 48 h of the *L. plantarum* fermentation, inulin showed the lowest pH value (4.08 ± 0.07), followed by HBb-Bps (4.13 ± 0.07) and FOS (4.14 ± 0.10). Meanwhile, HBb-Bp Honey maintained a significantly higher pH level (4.28 ± 0.02), close to the control (4.32 ± 0.09). Harmonically, the highest pH level of *L. lactis* fermentation was found in the culture with dextrose (4.37 ± 0.06). Other testing substances exhibited a final pH in the range of 4.02 to 4.18, indicating a higher acid production by these carbon sources. The culture media after 48 h of fermentations were subjected to reverse-phase HPLC for SCFA quantification. The production of acetic acid, propionic acid, and butyric acid was analyzed. Figure 6 demonstrates the production of total SCFA was highest with inulin, reaching 54.51 ± 3.52 mM for the *L. plantarum* fermentation, and 44.09 ± 4.12 mM for the *L. lactis* fermentation. The HBb-Bps and their conjugates produced lower amounts of SCFA in both bacterial strains, but higher than the control. Notably, in *L. lactis*, they generated significantly higher SCFA levels (25.41 ± 2.58 and 22.74 ± 3.31 mM) than the control (13.85 ± 1.64 mM). The pH changes and SCFA production data align to indicate that the HBb-Bps and their honey conjugates are effective prebiotics, promoting beneficial bacterial growth and SCFA production, which are essential for gut health.

## 4. Discussion

The purpose of this work was to investigate the potential health benefits of honeybee brood biopeptides and their conjugated products. This comprehensive research explored various functional properties, including antioxidant activities, inhibitory effects on key digestive enzymes, and their impact on the growth of probiotic bacteria and short-chain fatty acid production. This study was motivated by the growing interest in natural and functional ingredients in the food and pharmaceutical industries, leveraging the unique bioactive compounds found in honeybee brood.

Biopeptides are known for their antioxidant activities due to their ability to donate hydrogen atoms or electrons to free radicals, thus neutralizing them and preventing oxidative damage [35]. The specific amino acid composition and sequence of biopeptides play a crucial role in their antioxidant activity. For instance, peptides containing amino acids such as cysteine, methionine, histidine, and tryptophan are particularly effective antioxidants because these amino acids can directly scavenge free radicals or chelate metal ions, reducing the catalytic activity of the metal-catalyzed oxidation process. [36]. In this study, amino acid profiling of the honeybee brood hydrolysate revealed a distinctive composition, with valine constituting 17%, alanine also at 17%, and cysteine combined with phenylalanine making up 13%, along with glutamic acid at 13%. The other amino acids were present at levels below 10%. This amino acid profile differs significantly from that of the honeybee larva protein observed in our previous studies [13], which showed lysine at 8.6%, followed by leucine at 5.2%, and phenylalanine at 4.5%. The variation in amino acid content between these profiles is crucial, as it can significantly influence the bioactive properties of the peptides. For instance, the higher proportions of valine, alanine, and glutamic acid in HBb-Bps may enhance the antioxidant and ACE inhibitory activities, while different compositions in the honeybee larva protein might contribute to other health benefits. In the honeybee brood, a range of bioactive peptides with potent antioxidant properties has been reported [13,14,16]. Some works highlighted that honeybee brood contains significant amounts of vitamins, minerals, and other bioactive compounds that contribute to its overall antioxidant activity [16,37]. Additionally, studies demonstrated that protein hydrolysates derived from honeybee brood exhibited strong DPPH and ABTS radical scavenging activities [13,16].

In this study, HBb-Bps, primarily composed of bioactive peptides derived from honeybee brood, were prepared through fungal fermentation [28]. The peptides were then subjected to the Maillard reaction using a moist-dry heating process combined with a spontaneous aging technique, resulting in peptide–sugar conjugation. However, due to the degree of glycation being approximately 70%, the resulting mixtures also contained unreacted components from the Maillard reaction. This means that 30% of the peptides or sugars remained unmodified, leading to a final product that includes both conjugated and non-conjugated molecules. Nevertheless, the antioxidant of the HBb-Bps was significantly enhanced after conjugation with honey, glucose, and fructose, as evidenced by the increased inhibition rates in both the DPPH and ABTS assays. In addition, the antioxidant activity of HBb-Bps in this study was approximately 60% inhibition for the DPPH assay and 80% for the ABTS assay, which is notably higher than the findings of Morinova and Tchorbanov, 2011 [38], where honeybee-collected pollen hydrolyzed with plant-based enzymes, including bromelain, aminopeptidases, and proline iminopeptidase, exhibited a 42–46% inhibition in the DPPH assay. This comparison underscores the enhanced antioxidant potential of HBb-Bps, particularly when subjected to the Maillard reaction. This finding aligns with other studies demonstrating the enhanced antioxidant properties of Maillard reaction products [39]. The Maillard reaction leads to the formation of AGEs, which possess strong antioxidant properties. AGEs can donate electrons to neutralize free radicals more efficiently than their precursor peptides [40]. Moreover, the reaction may induce structural modifications in the peptides, such as increased hydrophobicity and the formation of new functional groups [41]. These changes can also enhance the peptides’ ability to interact with and neutralize free radicals. Another reason is that conjugation might create a synergistic effect due to their abilities. For instance, honey itself is known for its antioxidant properties due to its phenolic content [10,11]. When conjugated with the HBb-Bps, the antioxidant properties of both components are likely elevated.

This study evaluated the inhibitory activity of HBb-Bps and their conjugates on amylase and pancreatic lipase, key enzymes involved in carbohydrate and lipid digestion, respectively. Biopeptides can inhibit digestive enzymes through various mechanisms, including direct binding to the enzyme’s active site, competitive inhibition, and non-competitive inhibition by altering the enzyme’s conformation [42]. Specific amino acid residues in peptides, such as phenylalanine, tryptophan, tyrosine, and histidine, are often involved in these interactions [43]. Studies on biopeptides from different sources, such as fish, soybean, and oat bran, have shown that these peptides can effectively inhibit α-amylase and pancreatic lipase [7,44,45]. However, our findings indicated that the HBb-Bps exhibited low inhibitory effects on both enzymes, and these effects were reduced after conjugation with sugars through the Maillard reaction. Conjugation with sugars may induce significant conformation changes or create steric hindrance in the peptides, potentially reducing their ability to bind effectively to the active site [37,46]. In addition, some key amino acid residues may be modified or masked by the conjugated sugars, diminishing the peptide’s ability to inhibit the enzyme effectively [21,23,46]. Understanding these mechanisms is crucial for optimizing the use of biopeptides and their conjugates in foods and nutraceuticals with the aim of managing carbohydrate and lipid digestion.

The ACE plays a crucial role in regulating blood pressure by converting angiotensin I to vasoconstrictor angiotensin II. Inhibiting the ACE can help manage hypertension, making it a target for many therapeutic interventions [47]. We found that HBb-Bps, and honey, exhibited significant inhibitory activity against ACE. Moreover, when HBb-Bps were conjugated with honey and sugars via the Maillard reaction, the inhibitory activity was further enhanced. Protein hydrolysates from various sources have shown strong ACE inhibitory activity [17,42]. The presence of certain residues in peptides, including proline, lysine, and tryptophan, were determined to play a crucial role in ACE inhibitory activity [48]. Up to now, peptide-based ACE inhibitors derived from honeybee brood protein hydrolysate have been identified. Three novel ACE inhibitory peptides, including AVFPSIVGR, PPVLVFV, and PGKVHIT, purified from neutrase hydrolysates of honeybee brood were reported, with IC_50_ values of 6.44, 47.78, and 223.87 μM, respectively [17]. Consistently, hydrolysis of protein isolates from the bee pupae using Alcalase and Flavourzyme increased ACE inhibitory activity, with the highest inhibition reaching 66.71% (IC_50_ = 22.70 mg/mL). Further fractionation of the hydrolysates by molecular weight showed that the <1 kDa fraction had the highest ACE inhibitory activity at 96.98% with an IC_50_ of 0.66 mg/mL [49]. In this study, the fermentation process using *R. oligosporus* was employed to prepare the protein hydrolysate, which may result in different digestion outcomes and yield distinct peptide sequences. Further studies could be performed in the fractionation, purification, and sequence identification of these hydrolysates for supporting the application of honeybee brood hydrolysates as a functional ingredient in foods and nutraceuticals.

On the other hand, honey contains various bioactive compounds, including peptides, phenolic acid, and flavonoids, which can contribute to ACE inhibition [11,12]. Major Royal Jelly Proteins (MRJPs) and their derived peptides have demonstrated anti-inflammatory, immune-modulatory, antimicrobial, anticarcinogenic, hypotensive, hypolipidemic, growth-promoting, wound healing, and neuroprotective activities [50]. Peptides of Major Royal Jelly Protein 1 (MRJP1) from honeybee brood were identified as using an AHTpin server and QSAR models through silico methods. The result exhibited that the peptide sequence, EALPHVPIFDR, showed the strongest ACE inhibition by docking score [51]. Major phenolic acids found in honey, including caffeic acid, chlorogenic acid, and gallic acid, have been shown to have ACE inhibitory activity. Additionally, flavonoids, such as quercetin, kaempferol, luteolin, and apigenin, also exhibit potential ACE inhibitory activity [52].

Although honey contains various bioactive compounds with antioxidant activities and ACE inhibitory properties, this study found that the HBb-Bps conjugated with glucose and fructose exhibited enhanced antioxidant activity and ACE inhibition, Therefore, the increased antioxidant function and ACE inhibition observed in the HBb-Bp conjugates with honey likely results from both the intrinsic properties of honey and the effects of sugar conjugation.

Prebiotics are compounds that promote the growth and activity of beneficial gut bacteria [26,53]. This study assessed the prebiotic potential of the HBb-Bps and their conjugates by evaluating their impact on the growth of *L. plantarum* and *L. lactis* and the production of SCFA. The growth experiments showed that HBb-Bps and HBb-Bp Honey significantly promoted the growth of both probiotic strains, comparable to or better than that of traditional prebiotics like FOS and inulin. The pH measurements indicated that the HBb-Bps and their conjugates supported substantial fermentation activity, resulting in significant pH drops like the control and other prebiotics. The SCFA production analysis revealed that the HBb-Bps and their conjugates produced similar amounts of SCFA in both bacterial strains, which were higher than the control. Our findings are consistent with previous research on the prebiotic effect of protein hydrolysates and their Maillard reaction products. Studies have shown that such compounds can enhance the growth of probiotic bacteria and increase SCFA production, contributing to gut health [26,54]. The ability of HBp-Bps and their conjugates to act as prebiotics can be attributed to their peptide content, which serves as a nutrient source for beneficial bacteria. Moreover, certain biopeptides are reported to stimulate metabolic pathways in probiotics, enhancing their growth and activity [55]. The Maillard reaction further enhances the prebiotic properties by forming AGEs, involving functional properties, and creating synergistic effects with sugars [19,26]. These findings highlight the potential of HBb-Bps and their conjugates as effective prebiotics for functional food aimed at improving gut health.

## 5. Conclusions

This study provides comprehensive evidence of the health-promoting functions of the biopeptides derived from honeybee brood and their conjugated products. The enhanced antioxidant activities, the significant inhibitory effect on key enzymes, and, notably, the prebiotic properties of HBb-Bps highlight their potential in functional food and nutraceutical applications. The Maillard reaction played a crucial role in enhancing these biopeptides, underscoring the value of the HBb-Bps as natural, multifunctional ingredients. Considering the findings from this study, the HBb-Bps and their conjugated products could potentially be utilized in various industrial applications. However, future studies should include detailed structural analyses of HBb-Bps and their conjugates to better understand the conformational changes and modifications resulting from the Maillard reaction. This will provide deeper insights into their bioactive properties.

## Figures and Tables

**Table 1:** Changes in growth and pH throughout 48 h of fermentation of dextrose, HBb-Bps, HBb-Bps conjugated with honey, FOS, and inulin with *L. plantarum*, and *L. lactis*.

Bacteria/ Analysis	Carbon Source	Incubation Time (hours)							
		0	3	6	9	12	24	36	48
*L. plantarum*									
Growth (OD620)	Dextrose	0.04 ± 0.00 ^a^	0.15 + 0.01 ^a^	1.47 ± 0.12 ^b^	2.58 ± 0.12 ^b^	2.71 ± 0.09 ^a^	2.79 ± 0.08 ^a^	2.85 ± 0.08 ^b^	2.76 ± 0.04 ^c^
	HBb-Bps	0.05 ± 0.09 ^a^	0.22 + 0.11 ^a^	1.87 ± 0.02 ^a^	3.04 ± 0.24 ^a^	2.91 ± 0.03 ^a^	2.98 ± 0.09 ^a^	3.21 ± 0.00 ^a^	3.22 ± 0.02 ^a^
	HBb-Bp honey	0.05 ± 0.02 ^a^	0.22 + 0.04 ^a^	1.80 ± 0.04 ^a^	2.68 ± 0.21 ^b^	2.47 ± 0.17 ^b^	2.83 ± 0.11 ^a^	2.92 ± 0.02 ^b^	3.01 ± 0.04 ^b^
	FOS	0.04 ± 0.00 ^a^	0.15 + 0.01 ^a^	1.55 ± 0.11 ^b^	2.45 ± 0.11 ^b^	2.78 ± 0.05 ^a^	2.91 ± 0.06 ^a^	3.20 ± 0.10 ^a^	3.23 ± 0.06 ^a^
	Inulin	0.00 ± 0.01 ^a^	0.19 + 0.01 ^a^	1.53 ± 0.04 ^b^	2.53 ± 0.06 ^b^	2.82 ± 0.06 ^a^	3.00 ± 0.13 ^a^	3.05 ± 0.20 ^a^	3.09 ± 0.04 ^b^
pH	Dextrose	5.30 ± 0.04 ^a^	5.22 ± 0.05 ^a^	4.63 ± 0.07 ^a^	4.34 ± 0.02 ^a^	4.32 ± 0.08 ^a^	4.25 ± 0.03 ^a^	4.26 ± 0.04 ^a^	4.32 ± 0.09 ^b^
	HBb-Bps	5.42 ± 0.04 ^a^	5.32 ± 0.10 ^a^	4.79 ± 0.04 ^a^	4.53 ± 0.07 ^a^	4.38 ± 0.04 ^a^	4.16 ± 0.04 ^a^	4.16 ± 0.06 ^a^	4.13 ± 0.07 ^a^
	HBb-Bps honey	5.33 ± 0.05 ^a^	5.26 ± 0.03 ^a^	4.84 ± 0.04 ^a^	4.65 ± 0.11 ^a^	4.33 ± 0.05 ^a^	4.21 ± 0.07 ^a^	4.22 ± 0.10 ^a^	4.28 ± 0.02 ^b^
	FOS	5.38 ± 0.03 ^a^	5.32 ± 0.08 ^a^	4.68 ± 0.10 ^a^	4.42 ± 0.12 ^a^	4.27 ± 0.02 ^a^	4.12 ± 0.00 ^a^	4.19 ± 0.05 ^a^	4.14 ± 0.10 ^a^
	Inulin	5.31 ± 0.03 ^a^	5.34 ± 0.03 ^a^	4.73 ± 0.06 ^a^	4.45 ± 0.07 ^a^	4.22 ± 0.06 ^a^	4.08 ± 0.17 ^a^	4.06 ± 0.08 ^a^	4.08 ± 0.07 ^a^
*L. lactis*									
Growth (OD620)	Dextrose	0.04 ± 0.00 ^a^	0.34 ± 0.08 ^b^	1.13 ± 0.06 ^c^	2.12 ± 0.06 ^b^	2.65 ± 0.08 ^b^	2.98 ± 0.11 ^a^	2.75 ± 0.05 ^b^	2.56 ± 0.06 ^c^
	HBb-Bps	0.10 ± 0.00 ^a^	0.41 ± 0.08 ^b^	1.39 ± 0.06 ^b^	2.57 ± 0.06 ^a^	2.93 ± 0.08 ^a^	3.10 ± 0.11 ^a^	3.02 ± 0.05 ^a^	3.00 ± 0.06 ^a^
	HBb-Bps honey	0.05 ± 0.04 ^a^	0.56 ± 0.04 ^a^	1.71 ± 0.10 ^a^	2.42 ± 0.03 ^a^	2.84 ± 0.10 ^a^	3.13 ± 0.09 ^a^	2.98 ± 0.10 ^a^	3.01 ± 0.10 ^a^
	FOS	0.03 ± 0.00 ^a^	0.31 ± 0.05 ^b^	1.18 ± 0.10 ^c^	2.13 ± 0.06 ^b^	2.76 ± 0.07 ^a^	3.04 ± 0.08 ^a^	2.85 ± 0.07 ^a^	2.79 ± 0.06 ^b^
	Inulin	0.06 ± 0.05 ^a^	0.36 ± 0.05 ^b^	1.20 ± 0.10 ^c^	2.19 ± 0.06 ^b^	2.67 ± 0.07 ^b^	2.98 ± 0.08 ^a^	2.85 ± 0.07 ^a^	2.78 ± 0.06 ^b^
pH	Dextrose	5.33 ± 0.03 ^a^	5.26 ± 0.07 ^a^	4.89 ± 0.05 ^a^	4.51 ± 0.03 ^a^	4.50 ± 0.12 ^a^	4.41 ± 0.09 ^a^	4.34 ± 0.04 ^a^	4.37 ± 0.06 ^a^
	HBb-Bps	5.38 ± 0.04 ^a^	5.20 ± 0.05 ^a^	4.68 ± 0.07 ^a^	4.53 ± 0.05 ^a^	4.37 ± 0.06 ^a^	4.23 ± 0.07 ^b^	4.07 ± 0.05 ^b^	4.02 ± 0.10 ^b^
	HBb-Bps honey	5.36 ± 0.05 ^a^	5.17 ± 0.02 ^a^	4.76 ± 0.10 ^a^	4.63 ± 0.06 ^a^	4.41 ± 0.07 ^a^	4.24 ± 0.11 ^b^	4.18 ± 0.09 ^b^	4.15 ± 0.08 ^b^
	FOS	5.29 ± 0.05 ^a^	5.22 ± 0.04 ^a^	4.69 ± 0.12 ^a^	4.52 ± 0.04 ^a^	4.44 ± 0.01 ^a^	4.29 ± 0.11 ^a^	4.20 ± 0.05 ^b^	4.18 ± 0.10 ^b^
	Inulin	5.28 ± 0.04 ^a^	5.18 ± 0.07 ^a^	4.73 ± 0.03 ^a^	4.50 ± 0.08 ^a^	4.32 ± 0.01 ^a^	4.24 ± 0.02 ^b^	4.17 ± 0.10 ^b^	4.14 ± 0.05 ^b^

Data represented as mean ± SD (n = 3); lowercase letter (a–c) indicate values within each row with different superscript letters were significantly different (*p* < 0.05).

## Data Availability

The original contributions presented in this study are included in the article. Further inquiries can be directed to the corresponding authors.
